# Novel quantitative trait locus is mapped to chromosome 12p11 for left ventricular mass in Dominican families: the Family Study of Stroke Risk and Carotid Atherosclerosis

**DOI:** 10.1186/1471-2350-10-74

**Published:** 2009-07-23

**Authors:** Liyong Wang, Ashley Beecham, Marco R Di Tullio, Susan Slifer, Susan H Blanton, Tatjana Rundek, Ralph L Sacco

**Affiliations:** 1 Department of Human Genetics, Miami Institute for Human Genomics, Miller School of Medicine, University of Miami, Miami, FL, USA; 2 Department of Medicine, Columbia University, New York, NY, USA; 3 Department of Neurology and Epidemiology, Miller School of Medicine, University of Miami, Miami, FL, USA

## Abstract

**Background:**

Left ventricular mass (LVM) is an important risk factor for stroke and vascular disease. The genetic basis of LVM is unclear although a high heritability has been suggested. We sought to map quantitative trait loci (QTL) for LVM using large Dominican families.

**Methods:**

Probands were selected from Dominican subjects of the population-based Northern Manhattan Study (NOMAS). LVM was measured by transthoracic echocardiography. A set of 405 microsatellite markers was used to screen the whole genome among 1360 subjects from 100 Dominican families who had complete phenotype data and DNA available. A polygenic covariate screening was run to identify the significant covariates. Variance components analysis was used to estimate heritability and to detect evidence for linkage, after adjusting for significant risk factors. Ordered-subset Analysis (OSA) was conducted to identify a more homogeneous subset for stratification analysis.

**Results:**

LVM had a heritability of 0.58 in the studied population (p < 0.0001). The most significant evidence for linkage was found at chromosome 12p11 (MLOD = 3.11, empirical p = 0.0003) with peak marker at D12S1042. This linkage was significantly increased in a subset of families with the high average waist circumference (MLOD = 4.45, p = 0.0045 for increase in evidence for linkage).

**Conclusion:**

We mapped a novel QTL near D12S1042 for LVM in Dominicans. Enhanced linkage evidence in families with larger waist circumference suggests that gene(s) residing within the QTL interact(s) with abdominal obesity to contribute to phenotypic variation of LVM. Suggestive evidence for linkage (LOD = 1.99) has been reported at the same peak marker for left ventricular geometry in a White population from the HyperGEN study, underscoring the importance of this QTL for left ventricular phenotype. Further fine mapping and validation studies are warranted to identify the underpinning genes.

## Background

Stroke and cardiovascular diseases are of major public health concern, particularly among the rapidly growing Hispanic population [1]. Studies have shown that a positive family history is a strong predictor of stroke, even after adjustment for traditional risk factors, suggesting the existence of a substantial genetic component [2]. However, the genetic architecture of this common complex disease remains elusive. Given the extreme complexity of genetic and environmental contributions to stroke, evaluation of the precursor (risk) phenotypes, especially the quantitative ones, may reduce heterogeneity and increase statistical power for susceptibility gene discovery. Our Family Study of Stroke Risk and Carotid Atherosclerosis is uniquely designed to evaluate genetic contribution to cerebrovascular risk phenotypes, such as carotid intima-media thickness and left ventricular mass (LVM).

LVM is an important risk factor for stroke. In the Framingham Heart Study, the highest quartile of LVM was associated with a 2.7-fold increased risk of stroke when compared to the lowest quartile of LVM, after adjusting for age, sex, systolic blood pressure, treatment of hypertension and other established stroke risk factors [3]. We have also demonstrated that LVM is significantly associated with an increased risk of stroke and combined vascular events in a multi-ethnic cohort comprised predominately of Hispanics [4]. The adjusted hazard ratio for the combined endpoint of myocardial infarction, stroke, or vascular death was 1.34 per one standard deviation (SD) increase in LVM [95% CI 1.10 to 1.63].

As a quantitative trait, LVM is highly heritable. In the Framingham family study the heritability of LVM was estimated to be 0.24–0.32. [5] In the HyperGEN offspring study, the estimates of LVM heritability were 0.46 in African-Americans and 0.47 in Caucasians [6]. Heritability of LVM among Japanese subjects living in Hawaii was estimated to be 0.6 [7], similar to what we have seen in our sample of Caribbean Hispanics [8]. Consistent with a strong genetic influence, heritability estimates for LVM are often higher in twin studies than in sibling or family cohorts; the estimate is as high as 70% for monozygotic twins [9,10]. These studies suggested a substantial genetic contribution to LVM variations and encouraged an effort to map the quantitative trait loci (QTL) for this trait.

Previously, we have published a comprehensive heritability analysis on LVM as well as adjusted LVM measurements: LVM divided by body surface area or height; relative wall thickness; LV end-diastolic diameter; LV end-systolic diameter; interventricular septum thickness; and posterior wall thickness. The estimates of heritability for these LV measurments ranged from 0.23 to 0.65 after different adjustment for covariates, with LVM demonstrating the highest heritability (0.47 to 0.65 in different models) [8]. Therefore, we focused our QTL mapping effort on LVM in this study. In addition, we implemented the Ordered-subset Analysis (OSA) based on LVM-related covariates to strengthen linkage, narrow the critical linkage region and identify a more etiologically homogeneous sample that can be used to guide further fine mapping studies.

## Methods

### Subjects

The details of the family study design have been described elsewhere. [11] In brief, probands for the family study were selected from the population-based North Manhattan Study (NOMAS). In order to maximize the genetic component in the families for stroke risk phenotypes and thereby enhance our chance to map susceptibility genes, we assembled a high-risk Caribbean Hispanic family dataset using the following criteria to define a qualifying proband: (1) reporting a sibling with a history of myocardial infarction or stroke; or (2) having 2 of 3 quantitative risk phenotypes (maximal carotid plaque thickness, left ventricular mass, or homocysteine level above the 75th percentile in the NOMAS cohort). The majority of the probands (80%) were recruited based on the first criteria. Families of the eligible probands were considered for enrollment if the proband was able to provide a family history, obtain the family member's permission for the research staff to contact them, and had at least two additional first-degree relatives able to participate. After the proband was contacted, the study coordinator followed up with the relatives to explain the study and solicit participation. Although the proband was identified in Northern Manhattan, we enrolled family members in New York at Columbia University and in the Dominican Republic at the Clinicas Corazones Unidos in Santo Domingo. All subjects provided informed consent to participate in the study and the study was approved by the Institutional Review Boards of Columbia University, University of Miami, and the National Bioethics Committee and the Independent Ethics Committee of Instituto Oncologico Regional del Cibao in the Dominican Republic (DR).

Demographic, socioeconomic and risk factor data were collected through direct interview of each family member based on instruments developed in NOMAS [12]. Extensive questionnaires regarding hypertension, diabetes, smoking, alcohol use, and physical activity were completed using systematic instruments. Measurements of anthropometrical indices including height, weight, hip and waist measures and skin-fold thickness were obtained, as were serial blood pressures. Fasting blood was collected and processed for lipids (total cholesterol, LDL, triglyceride, HDL), glucose, electrolytes, and creatinine. Storage of buffy coats and extraction of DNA was done by the Columbia University Genome Center.

### Phenotyping

Baseline transthoracic echocardiography was done on 1360 Dominican family members and probands in the Family Study of Stroke Risk and Carotid Atherosclerosis. Standard two-dimensional echocardiography, including color-Doppler flow study was performed according to the guidelines of the American Society of Echocardiography [13]. Special attention was paid to obtaining high quality parasternal long axis views of the left ventricle, from which left ventricular end-diastolic diameter (LVDD), left ventricular end-systolic diameter (LVSD), interventricular septum (IVS), and posterior wall thickness (PWT) were derived [14]. Sonographer performance was monitored quarterly after review of a random sample for technical adequacy of the images. Readers were blinded to familial relationships and vascular risk factors. Inter-observer variability for the variables of interest ranged between 8% and 10%. LVM was calculated according to the modified American Society of Echocardiography (ASE) formula: LVM = 0.8 [1.04 (LVDD + IVS+PWT)^3 ^- (LVDD)^3^] +0.6. [15].

### Genotyping and Quality Control

DNA was sent to the Center for Inherited Disease Research (CIDR) for genotyping. A set of 405 microsatellite markers were genotyped at an average interval of 10 centimorgan (cM) across the genome. Autosomal microsatellite genotypes were used to verify and adjust family structure using the program PREST. [16] In order to do so, the Maximized Log-Likelihood Ratio (MLLR) test statistics were computed to compare the putative relationship between pairs of individuals to those constructed based on the autosomal genotypes. Relationships with a p-value for the MLLR test of < 0.000001 in a consistent manner across the family were considered in error. Estimated kinship coefficients and identity by descent (IBD) estimates were used to rearrange family structure as needed. Finally, family structure was confirmed by running PREST again. Mendelian error checking was performed on the final family structure using Pedcheck [17].

### Statistics

Heritability was evaluated using a pedigree-based maximum-likelihood method implemented in SOLAR. [18] Variance components methodology as implemented in SOLAR has emerged as a powerful method to detect linkage of quantitative traits and was used to calculate two-point and multipoint LOD scores [19,20]. Ascertainment correction was automatically incorporated in the linkage analysis. First, marker-specific IBD were computed using the David and Weeks Monte Carlo algorithm for each marker. After marker-specific IBDs were merged together into one single file, multipoint IBDs were calculated using a 1 cM grid. Empirical p-values for LOD scores were calculated based on 10,000 replicates in which a fully-informative marker, unlinked to LVM, was simulated and used to compute possible LOD scores. Since SOLAR requires that quantitative traits be normally distributed and properly scaled, LVM measurements were natural-log transformed and multiplied by 10. To ensure normality, two outliers, whose LVM measurements were beyond three standard deviations from the mean, were dropped from further analysis.

To decide the covariates that should be included in estimating heritability and calculating LOD scores, a polygenic covariate screening implemented in SOLAR was run to screen age, sex, smoking, diabetes, dyslipidemia, hypertension, and body mass index (BMI). Interaction between age and sex was automatically included in SOLAR. A permissive threshold of p < 0.1 was used for this evaluation to allow for inclusion of any potentially significant covariates. Hypertension was defined as reported history of high blood pressure, systolic blood pressure ≥ 140 mmHg, diastolic blood pressure ≥ 90 mmHg, or use of antihypertensive medication. Smoking was defined as never versus ever. Dyslipidemia was defined as a history of hyperlididemia or total cholesterol greater than 240 mg/dL.

For the OSA, trait-related quantitative covariates were used to rank families for a more homogeneous subset [21]. LOD scores calculated in the overall linkage analysis for each family were used as input. Families were sequentially introduced into linkage analysis by the order of increasing or decreasing covariate values until the maximum evidence for linkage was achieved. A permutation procedure was implemented to generate an empirical p-value for the significance of the increase in the LOD score from the overall dataset to OSA identified subset. Specifically, 10,000 random family orderings were permuted and the maximum LOD scores from each of the random orderings were compared with the OSA results to derive the empirical p-value. All statistical analyses were performed using SAS v9.1.3 (SAS Institute Inc., Cary, NC) unless specified otherwise.

## Results

Overall, 110 families with high vascular disease burden (Dominican Republic 100, Puerto Rico 4, Cuba 2, Ecuador 2, Nicaragua 1, and Colombia 1) were enrolled in our family study. Seventy percent of the subjects were enrolled in Northern Manhattan and 30% in the Dominican Republic. In order to reduce population stratification, we restricted our QTL mapping to the 100 Dominican families, which was composed of 2182 individuals. The mean family size was 22 ± 11 with median of 20, and range of 4–87. LVM measurements and DNA samples were available for 1360 subjects from the 100 Dominican families. Among the 1360 individuals, there were 1207 sib pairs, 362 half-sib pairs, and 1713 avuncular pairs. With our dataset, we had over 80% power to detect QTLs for traits with heritability estimates greater than 0.18 at a LOD score threshold of 2.0. The mean age of the study cohort was 46 years. The mean LVM was 174.85 ± 56.14 (Table 1). Men had significantly greater mean LVM than women (201.42 vs 158.15, p < 0.0001).

**Table 1:** Sociodemographic, vascular risk factors, and left ventricular mass measurements

	**Males**		**Females**		**Total**	
	**(N = 525)**		**(N = 835)**		**(N = 1360)**	
			
	**n**	**%**	**n**	**%**	**n**	**%**
Hypertension	200	38.10	340	40.72	540	39.71
Diabetes	77	14.67	109	13.05	186	13.68
Coronary artery disease*	80	15.24	164	19.64	244	17.94
Ever smoking	217	41.33	256	30.66	473	34.78
≥ High School Education	266	50.67	399	47.78	665	48.9
Enrolled in the DR	164	31.24	262	31.38	426	31.32
						
	**Mean ± SD**		**Mean ± SD**		**Mean ± SD**	
Age	44.59 ± 17.21		47.10 ± 17.24		46.13 ± 17.26	
Body mass index (kg/m2)	28.44 ± 5.34		28.98 ± 6.06		28.77 ± 5.80	
Waist circumference (inch)	37.99 ± 5.18		35.57 ± 5.60		36.50 ± 5.57	
Triceps skinfold thickness (mm)	21.12 ± 10.89		30.68 ± 10.62		27.00 ± 11.69	
Fast glucose (mg/dl)	94.92 ± 44.12		89.38 ± 31.62		91.52 ± 37.04	
Total cholesterol (mg/dl)	183.31 ± 44.25		186.97 ± 40.58		185.56 ± 42.05	
LDL (mg/dl)	110.76 ± 35.64		110.52 ± 34.96		110.61 ± 35.21	
HDL (mg/dl)	44.80 ± 11.77		53.34 ± 13.55		50.05 ± 13.55	
Triglyceride (mg/dl)	141.95 ± 116.77		115.46 ± 67.09		125.66 ± 90.43	
Systolic blood pressure (mmHg)	124.18 ± 18.05		120.35 ± 20.69		121.82 ± 19.80	
Diastolic blood pressure (mmHg)	79.05 ± 10.84		75.78 ± 10.38		77.04 ± 10.68	
Left ventricular mass	201.42 ± 60.88		158.15 ± 45.65		174.85 ± 56.14	

*Coronary artery disease was defined as having a history of bypass surgery, angioplasty, or myocardial infarction.

The polygenic covariate screening identified age-sex interaction, sex, ever smoking, systolic blood pressure (SBP), and BMI as significant covariates. Heritability and LOD scores were calculated adjusting for these covariates. The significant covariates together explained 0.33 of the total LVM variance. The heritability estimate of LVM is 0.58 (p < 0.0001) for the remaining variance of LVM after adjusting for the significant covariates.

**Figure 1:**
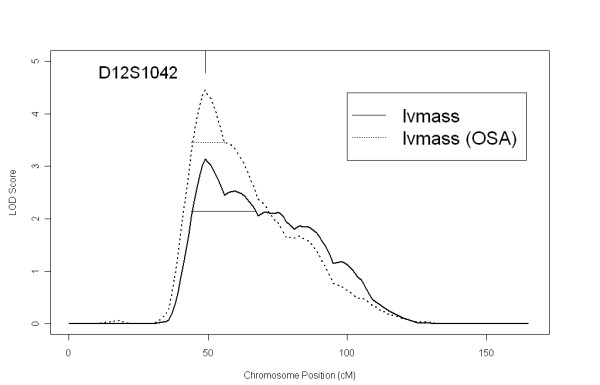
**Multipoint linkage plots for left ventricular mass on chromosome 12 in overall families and subset families defined by ordered-subset analysis on waist circumference**. Maximum multipoint LOD scores were plotted along the chromosomal position in centimorgans (cM) on chromosome 12. Horizontal lines indicate the one-LOD unit down supporting intervals. All 100 families were used in the overall analysis and the LOD score is depicted by solid line. In the ordered-subset analysis, the families were ranked by mean waist circumference from high to low and sequentially introduced to linkage analysis until the maximum LOD score was achieved. 54 families with the highest waist circumference were used in the final analysis and the LOD score is depicted by the dashed line.

To localize the genetic loci affecting LVM variations, we completed a QTL mapping on LVM. This analysis revealed a significant region on chromosome 12p11 with peak marker at D12S1042 (MLOD = 3.11, empirical p value = 0.0003) (Figure 1). The one-LOD unit down critical interval extends from approximately 25 Megabase (Mb) to 51 Mb on chromosome 12, encompassing 181 annotated genes. No evidence for linkage (LOD > 1.5) was found in any other region through the genome (data not shown). Estimation of the locus specific heritability is 0.25 at D12S1042 and this locus accounted for 43% of the total heritability of LVM in our dataset.

To better characterize the prominent linkage region on 12p11, we performed OSA to further reduce phenotypic heterogeneity. In this analysis, trait-related quantitative covariates were used to rank families for a more homogeneous subset. We chose SBP, BMI, and waist circumference (WC) as the covariates to order families because high blood pressure and obesity, especially elevated waist circumference (WC), are major risk factors that lead to an increase of LVM in general population [22-24]. We ordered families by family average SBP, BMI, and WC. OSA results based on high to low (H-L) or low to high (L-H) ranking of the three covariates are listed in Table 2. The OSA maximum LOD score was increased in all H-L rankings (LOD = 3.81 to 4.45) but was not changed in the L-H rankings (LOD = 3.14 to 3.40), when compared to the overall analysis. Permutation tests were performed to evaluate the significance of increases in LOD scores. Among all the OSA ranking strategies that led to a higher LOD score, ordering families by decreasing average WC significantly increased the evidence for linkage (LOD = 4.45) (p = 0.0045 for increase in LOD score). In addition to significantly strengthening the linkage, this ranking strategy also greatly reduces the one-LOD unit down supporting interval size from 26 Mb to 14 Mb (Figure 1). The narrowed critical linkage region extends from 25 Mb to 39 Mb on chromosome 12 harboring 55 annotated genes. The OSA defined subset included 54 families with high WC. The average WC was 38.4 inches for the OSA subset families and 34.4 inches for the rest of the families (Table 2).

**Table 2:** Ordered-Subset analysis results on chromosome 12 p11.

**Covariate**	**Position (cM)**	**Maximum OSA Lod**	Empircal P value*	**Number of Subset Families**	**Mean Covariate Value (SD) in subset**	**Mean Covariate Value (SD) in other families**
WC (H to L)†	49	4.45	0.0045	54	38.4 (1.5)	34.4 (1.5)
WC (L to H)†	49	3.14	1	100	36.5 (2.5)	NA
SBP (H to L)	49	3.81	0.1075	84	124.2 (6.9)	112.6 (3.0)
SBP (L to H)	49	3.40	0.4228	94	121.1 (6.1)	141.5 (4.5)
BMI (H to L)	49	3.89	0.0759	78	30.0 (2.2)	25.3 (1.2)
BMI (L to H)	49	3.16	0.7716	98	28.8 (2.7)	35.5 (1.2)

† H to L: high to low; L to H: low to high; * p value is based on 10,000 permutations.

## Discussion

Using well-characterized, extended Dominican Republic families, we mapped a novel QTL for LVM on chromosome 12p11. This QTL influences LVM independent of other risk factors such as gender, age, hypertension, BMI, and smoking. By implementing OSA to reduce phenotype heterogeneity, we have greatly strengthened the evidence for linkage, narrowed the linkage region and identified a sub-phenotype that has concentrated genetic effects from chromosome 12p11. Our findings provide a well-defined chromosome region and phenotype for future validation and fine mapping studies to localize genes controlling LVM.

The majority of genetic studies on LVM phenotype to date have focused on candidate genes derived from biological pathways that have impact on the LVM phenotype, such as genes implicated in myocardial cell growth/remodeling, calcium homeostasis, extracellular matrix, hemodynamic load, and blood pressure regulation. Polymorphisms in several genes have been associated with LVM, such as the 825C/T polymorphism in the G protein beta subunit (GNB3) gene, [25-27] polymorphism in the beta-1 adrenergic receptor (beta-1 AR) gene, [28], the -344C/T polymorphism and a gene conversion in intron 2 in the aldosterone synthase gene, [29,30] and the polymorphism in the angiotensin-converting-enzyme (ACE) gene. [31] However, the association results have been conflicting [10,32]. Recently, a meta-analysis of 485 reports concluded that the -344C/T polymorphism in the aldosterone synthase gene is not significantly associated with LVM [33].

Other approaches to identify the underpinning genes for a disease-associated trait are unbiased genomic approaches, such as genome-wide linkage and association studies. These studies do not require *a prior*i knowledge on the molecular basis of phenotypic variations and have advantages in identifying unknown/unexpected genes and pathways for complex traits. Previously, two genome-wide studies of LVM as a quantitative trait have been reported. Both of them were conducted in Caucasian populations [34,35]. One was completed in white British families with hypertension and another one was conducted in families from the Framingham Heart Study. In the British study, suggestive evidence for linkage was found on chromosome 5p (MLOD = 1.6) for echocardiography-derived LVM (echo-LVM), and on chromosome 7q (MLOD = 1.7) and 12q (MLOD = 2.2) for electrocardiography-derived LVM (ECG-LVM) [34]. In the Framingham Heart Study, 100,000 of single nucleotide polymorphisms (SNPs) were tested for association with LVM. None met the genome-wide significance level. Three SNPs with a nominal p-value less than 10^-5 ^were found on chromosomes 2, 6, and 11. One SNP is located within a heat shock protein gene *HSPA8*; the other two SNPs are not located in or near known genes. Linkage analysis revealed strong evidence for linkage on chromosome 5q (MLOD = 4.4) and suggestive evidence for linkage on chromosomes 1q and 8q (MLOD = 2.4).

There is no overlap among the QTLs delimited in the British, the Framingham Heart, and the current studies. One possible explanation is that the unique ethnic population in our study may have a different genetic basis for LVM than Caucasians analyzed in the other two studies. However, the lack of replication even between the British and Framingham studies as well as the conflicting candidate gene studies, underscore the challenges in genetic analysis of complex traits: the genetic basis is likely to be complex and heterogeneous, involving multiple genes each with moderate effect and interaction with environmental factors. Within a given dataset, only one or two loci with the strongest effect in that population can be detected. In our study, the 12p11 QTL only accounts for less half of the total heritability of LVM. Other loci that have smaller effect sizes are still at large due to the limited power of the current dataset. Future studies in a similar population, including meta analyses are needed to catalogue all the genetic causes for the inter-individual LVM variations.

To reduce heterogeneity, stratification analysis based on a trait-related variable has been proposed in genetic studies of complex traits. The OSA ranks families by a trait-related variable and sequentially introduces the families to the linkage analysis until the maximum evidence of linkage is observed [36]. The subset defined by OSA is likely to represent a more etiologically homogeneous sample and thus increases the statistical power. Furthermore, the more homogeneous phenotype defined in the OSA subset provides insight to guide further analysis to pinpoint the genetic variants contributing to the trait. For example, in the GENECARD family study of coronary artery disease (CAD), OSA has identified a subset of families with very early age-of-onset (mean age-of-onset = 37.8 years) with strong evidence for linkage on chromosome 7p (LOD = 4.2). This finding was used to guide further association studies in subjects with early onset CAD and led to the discovery of the Neuropeptide Y gene polymorphism as genetic risk factor for atherosclerosis [37].

Using OSA, we found that the linkage signal on chromosome 12p11 is mainly from families with high WC. To exclude the possibility that we mapped a locus for WC instead of LVM, we performed a linkage analysis on WC and found no evidence suggesting that the signal on 12p11 is driven by WC (LOD = 0 at D12S1042 for WC). Therefore, the 12p11 linkage is a bona fide QTL for LVM and genes under this QTL interact with visceral obesity to influence inter-individual LVM variations. Adipose tissue is an active organ that secretes a variety of bioactive peptides. Several proteins of the renin angiotensin system, an essential regulatory axis for blood pressure, are expressed in adipose tissue. [38] Among them, angiotensinogen (AGT), angiotensin receptors type I (AT1), and ACE have higher expression in visceral than subcutaneous adipose tissue. Indeed, plasma AGT and plasma ACE activity are higher in obese individuals. [38] One possible scenario is that the candidate gene(s) in 12p11 QTL interact(s) with the bioactive peptides produced in visceral adipose tissue, such as ACE, to affect LVM. This interaction could confound phenotype-genotype correlations and should be taken into account in future fine-mapping studies.

The OSA based on BMI did not significantly enhance the linkage evidence despite that BMI and WC are correlated measurements, suggesting that WC provided additional information to BMI in the analysis of LVM. Consistently, recent reports from Rodrigues et al. [39] and Tsioufis CP et al. [40] showed that WC is an important predicator of LV hypertrophy and LV diastolic dysfunction. Furthermore, the impact of WC on LV structure and function seemed to be especially strong in women in those two studies. This gender specific effect should be considered in future studies in population-based cohort. The OSA based on SBP did not identify any subset that was significantly different from the randomly ordered families. However, our study is limited by the single clinic measurement of blood pressure and antihypertensive treatment. Blood pressure variation throughout the day is better predictor of left ventricular hypertrophy than a single clinic measurement, and antihypertensive treatment may reduce left ventricular hypertrophy depending on how long the patient has been treated. These could obscure the correlation between blood pressure and LVM.

It is worth noting that in the HyperGEN study suggestive evidence for linkage (LOD = 1.99) for LV geometry was found at the same peak marker (D12S1042) for LV mass in our study [41]. LV geometry is a combination of LV mass and relative wall thickness (RWT) of LV, with LV mass contributing to forming the different LV geometric patterns and RWT being an index of concentric geometry. Although the LV measurement is not exactly the same examined in our study, the encouraging LOD score at the same chromosomal location as found in the HyperGEN study corroborates our findings of chromosome 12p11 as a potential locus for LVM due to similarity between LV mass and LV geometry. Nonetheless, further validation studies on LVM are desired to confirm the QTL.

Our success in mapping a strong QTL relies on the strength of our study design. We focused on one ethnic group and used a family study design to minimize the effects of population substructure. All the families have high vascular disease burden and detailed systematic measurements of the quantitative phenotype. The extended family structure (average family size of 20) in our dataset provides unprecedented information content and statistical power. The QTL mapping approach also offers additional statistical advantages over discrete traits. A major limitation of our study is that we did not pinpoint the causal genetic variations that lead to increased LVM. This study is only the first stage to elucidate the genetic basis for LVM. Looking through all the genes under the 12p11 linkage peak did not find any gene that would have an obvious effect on LVM based on the current knowledge. However, the molecular basis of increased LVM is not clear and many genes' functions are not fully understood. We plan to perform fine mapping study across the linkage peak to narrow the critical region and identify the candidate gene in future studies.

Most genetic studies of cardiovascular disease have focused on non-Hispanic populations [42-46] with a few exceptions such as the San Antonio Family Heart Study and the Multi-ethnic Study of Atherosclerosis (MESA) [47,48]. Our family study provides essential data to fill the gaps in our knowledge of the genetics of cerebrovascular risk phenotypes in minority populations.

## Conclusion

With the unbiased genome-wide approach, we mapped a novel QTL for LVM, a significant risk phenotype for stroke and vascular events. The strong evidence for linkage on chromosome 12p11 warrants further validation and fine mapping efforts. In addition, our family study provides unique data on the genetics of cerebrovascular risk phenotypes in an understudied minority population.

## Competing interests

The authors declare that they have no competing interests.

## Authors' contributions

LW: planned the analysis, interpreted data, draft the manuscript. AB: analyzed the data and participated in the interpretation of data. MRD: ascertained and phenotyped the subjects, assisted in study design, revised the manuscript for important intellectual content. SS: designed the analysis, analyzed the data, and participated in the interpretation of data. SHB: designed the analysis, interpreted data, and critically revised the manuscript. TR: ascertained and phenotyped the subjects, assisted in study design, and critically revised the manuscript. RS: conceived the overall study, secured funding, and critically revised the manuscript. All authors have read and approved the content of the manuscript.

## Pre-publication history

The pre-publication history for this paper can be accessed here:


